# Inhibiting Histone Deacetylases in Human Macrophages Promotes Glycolysis, IL-1β, and T Helper Cell Responses to *Mycobacterium tuberculosis*

**DOI:** 10.3389/fimmu.2020.01609

**Published:** 2020-07-23

**Authors:** Donal J. Cox, Amy M. Coleman, Karl M. Gogan, James J. Phelan, Cilian Ó Maoldomhnaigh, Pádraic J. Dunne, Sharee A. Basdeo, Joseph Keane

**Affiliations:** Trinity Translational Medicine Institute, St. James's Hospital, Trinity College, The University of Dublin, Dublin, Ireland

**Keywords:** glycolysis, tuberculosis, human alveolar macrophage, T cell, immunomodulation, HDACi, SAHA, Vorinostat

## Abstract

Tuberculosis (TB) is the leading infectious killer in the world. *Mycobacterium tuberculosis* (Mtb), the bacteria that causes the disease, is phagocytosed by alveolar macrophages (AM) and infiltrating monocyte-derived macrophages (MDM) in the lung. Infected macrophages then upregulate effector functions through epigenetic modifications to make DNA accessible for transcription. The metabolic switch to glycolysis and the production of proinflammatory cytokines are key effector functions, governed by epigenetic changes, that are integral to the ability of the macrophage to mount an effective immune response against Mtb. We hypothesised that suberanilohydroxamic acid (SAHA), an FDA-approved histone deacetylase inhibitor (HDACi), can modulate epigenetic changes upstream of the metabolic switch and support immune responses during Mtb infection. The rate of glycolysis in human MDM, infected with Mtb and treated with SAHA, was tracked in real time on the Seahorse XFe24 Analyzer. SAHA promoted glycolysis early in the response to Mtb. This was associated with significantly increased production of IL-1β and significantly reduced IL-10 in human MDM and AM. Since innate immune function directs downstream adaptive immune responses, we used SAHA-treated Mtb-infected AM or MDM in a co-culture system to stimulate T cells. Mtb-infected macrophages that had previously been treated with SAHA promoted IFN-γ, GM-CSF, and TNF co-production in responding T helper cells but did not affect cytotoxic T cells. These results indicate that SAHA promoted the early switch to glycolysis, increased IL-1β, and reduced IL-10 production in human macrophages infected with Mtb. Moreover, the elevated proinflammatory function of SAHA-treated macrophages resulted in enhanced T helper cell cytokine polyfunctionality. These data provide an *in vitro* proof-of-concept for the use of HDACi to modulate human immunometabolic processes in macrophages to promote innate and subsequent adaptive proinflammatory responses.

## Introduction

Tuberculosis (TB) is the world's leading infectious killer (1). Current treatment regimens require many months of multiple drugs which are often not completed (2). Moreover, there is a significant rise in the incidence of multi-drug resistant TB, thus there is an urgent need for new therapeutic and vaccine strategies (1).

*Mycobacterium tuberculosis* (Mtb), the bacteria that causes TB, is phagocytosed by resident alveolar macrophages (AM), and infiltrating monocyte-derived macrophages (MDM) which then upregulate bactericidal effector functions. These effector functions are governed by changes in chromatin structure and gene transcription (3–5). DNA is tightly packed and condensed around histones, which inhibits access to genes. One of the major regulators of gene transcription is the acetylation status of histones which is controlled via two families of enzymes; histone acetyl transferases (HAT) and histone deacetylases (HDAC) (6). In general, acetylation of histones opens the packed DNA to make it accessible for transcription and therefore active, whereas HDAC close the DNA by removing acetyl groups from histones. Mtb infection can target host HDAC to modulate the immune response (7, 8). In keeping, HDAC inhibitors (HDACi) are being explored for their ability to modulate the development of TB (3, 9, 10).

We have previously established that glycolytic metabolism has a critical role in human AM function during Mtb infection (11). Metabolic changes and the switch to a pro-inflammatory macrophage phenotype are governed by epigenetics (12–16), and since previous studies have suggested that HDACi modulate macrophage function (3, 17–22), we sought to determine whether the pan HDACi suberanilohydroxamic acid (SAHA; also known as Vorinostat) could modulate macrophage function during Mtb infection.

Macrophages can direct memory T cell responses in the lung. T cells, activated in the lymph nodes, traffic to the lung, and require restimulation by tissue-resident antigen presenting cells (APC). Moreover, the suppressive lung environment promotes regulatory T (Treg) cells (23) and dampens effector T cells. We hypothesised that inhibiting histone deacetylases (HDAC) may improve macrophage responses to Mtb and subsequently elicit T cells with enhanced effector function.

We examined the ability of the FDA-approved HDAC inhibitor, SAHA, to modulate early clearance events in macrophages infected with Mtb. SAHA increased glycolysis in human macrophages early in the response to stimulation with Mtb. Furthermore, SAHA increased IL-1β and decreased IL-10 production in human AM and MDM. Infected macrophages treated with SAHA enhanced T helper (Th) cell responses, resulting in increased IFN-γ and GM-CSF production.

## Materials and Methods

### MDM Cell Culture

Peripheral blood mononuclear cells (PBMC) were isolated from the buffy coats of healthy donors (Irish Blood Transfusion Services) or from the venous blood of Interferon Gamma Release Assay (IGRA) positive antibiotic-treated, otherwise healthy individuals attending St. James's Hospital respiratory outpatients' clinic (as approved by the Ethics Board), by density-gradient centrifugation over Lymphoprep (StemCell Technologies). Cells were washed, resuspended at 2.5 × 10^6^ PBMC/ml in RPMI (Gibco) supplemented with 10% AB human serum (Sigma-Aldrich) and 1 ml of cell suspension was plated on to non-treated 24-well-tissue culture plates (Costar). Cells were maintained in humidified incubators for 6–7 days at 37°C and 5% CO_2_. Non-adherent cells were removed by washing every 2–3 days. The purities of MDM were assessed by flow cytometry and were routinely >95% pure. Approximately 10% of the PBMC differentiate into MDM. PBMC were also cryopreserved for co-culture assays.

### AM Acquisition and Culture

Human AM were retrieved at bronchoscopy, as approved by the Ethics Board of St. James's Hospital, and previously reported by us (24). All donors were patients undergoing clinically indicated bronchoscopy and written informed consent for retrieving additional bronchial washings for research was obtained prior to the procedure. Patients were not remunerated for participation in this study. Exclusion criteria included age under 18 years, inability to provide written informed consent or a known (or ensuing) diagnosis of malignancy, sarcoidosis, HIV, or Hepatitis C. Patients undergoing biopsy as part of bronchoscopy were also excluded. For the seven subjects recruited to this study, clinical indications for bronchoscopy included haemoptysis (3/7), cough (2/7), bronchitis (1/7), and atelectasis (1/7). Two patients were non-smokers, while five were either current or ex-smokers, of which two had diagnosed chronic obstructive pulmonary disease.

Sample acquisition during bronchoscopy: Conscious sedation was achieved using intravenous midazolam and lignocaine gel was administered to the nostril. Flexible video-bronchoscope was inserted through the nostril and advanced to the level of the vocal cords by posterior approach. Further lignocaine spray was administered prior to and subsequent to traversing the vocal cords. Following routine bronchoscopy, the bronchoscope was wedged in the right middle lobe bronchus. A total of 180 ml of sterile saline was administered as 60 ml boluses via a connector inserted into the bronchoscope and aspirated within 5–10 s under low suction. The bronchoalveolar lavage fluid (BALF) was then transported directly to the laboratory for AM isolation. Pre- and post-bronchoscopy patient care was not altered by participation in the study. The procedure was prolonged by ~12 min.

Cells were seeded at 5 × 10^5^ cells per ml (2.5 × 10^5^ per well in a 48 well-plate) in RPMI (Gibco) supplemented with 10% FBS (Gibco), fungizone (2.5 μg/ml; Gibco) and cefotaxime (50 μg/ml; Melford Biolaboratories). Cells were incubated for 24 h at 37°C and 5% CO_2_ before washing to remove non-adherent cells. Adherent cells (predominantly AM) were then used for experiments.

### Mycobacterial Culture and Infection of Macrophages

Mtb H37Ra was obtained from The American Type Culture Collection (ATCC 25177™; Manassas, VA) and propagated in Middlebrook 7H9 medium supplemented with ADC (Beckton Dickinson), to log phase. Irradiated H37Rv (iH37Rv) was gifted by BEI Resources. The multiplicity of infection (MOI) and donor variation in phagocytosis of Mtb was adjusted for by Auramine-O staining, as previously described (25). MDM or AM were plated on 8-well Lab-Tek chamber slides (Nunc). Macrophages were infected with a range of bacterial concentrations for 3 h before extracellular bacteria were thoroughly washed off. Cells were fixed with 2% PFA, stained with Hoechst 33342 (10 μg/ml; Sigma-Aldrich), and rapid Auramine O staining set (Scientific Device Laboratory Inc). The numbers of bacilli per cell were counted in at least 30 fields of vision per well on a fluorescent microscope (Olympus IX51). The volume of bacterial suspension required to yield an MOI of 1–10 bacteria per cell (~70% of macrophages infected with at least 1 bacillus; median number of 5, average 4–6, mode 2–4 bacilli per cell, as plotted in Supplemental Figures 2D,E) was determined. Macrophages were infected in the presence of SAHA (20 μM) or equivalent volumes of vehicle control (0.1% DMSO; both Sigma-Aldrich). After 3 h extracellular bacteria were washed off and macrophages were incubated as indicated. Uninfected macrophages were assayed in parallel as controls.

### Seahorse Analysis of Metabolic Function

The impact of SAHA on macrophage metabolic function was assessed using the Seahorse XFe Analyzer (Agilent). PBMC were isolated from healthy control buffy coats and MDM were adherence purified in non-treated 6 well-plates (Costar; 2.5 × 10^6^ cells/ml, 4 ml/well). MDM were gently scraped, counted using trypan blue, and seeded onto Seahorse plates (1 × 10^5^ viable cells per well, seeded in low volume for 2 h to facilitate adherence then topped up to 500 μl and allowed to rest overnight in a humidified incubator at 37°C and 5% CO_2_ before analysis on the Seahorse XFe24 Analyzer. The extracellular acidification rate (ECAR) and the oxygen consumption rate (OCR), surrogates for glycolysis, and oxidative phosphorylation, respectively, were measured approximately every 10–20 min for 600 min. Human MDM were stimulated by injecting iH37Rv into wells through the Seahorse Analyzer ports 30 min after initiation. DMSO or SAHA were injected into corresponding wells 2 h post-infection. ECAR and OCR were tracked in real time for the following 10 h. Alternatively, the ECAR and OCR of MDM were examined 24 h post infection and treatment with SAHA or DMSO.

### Macrophage Assays

The concentrations of IL-1β, IL-10 (BioLegend ELISA Max Deluxe kits), and TNF (Invitrogen ready-set-go kit) present in the supernatants were quantified by ELISA, according to the manufacturer's protocol.

To assess the impact of SAHA (20 μM; Sigma-Aldrich) on the viability of human MDM, cells were stained with propidium iodide (PI), and Hoechst 33258 and 33342 at the indicated time-points post-infection with Mtb or treatment with cycloheximide (positive control; 50 μg/ml). Cells were incubated for 30 min at room temperature in the dark and analysed on the Cytell Cell Imaging System (GE Healthcare Life Sciences).

The effect of SAHA on macrophage phagocytosis was assessed using fluorescent latex beads (2 μm; Sigma-Aldrich); healthy control human MDM were treated with fluorescent latex beads (125 μg/ml) for 60 min at 37°C in the presence of SAHA or vehicle control. Cells were thoroughly washed, fixed with 2% paraformaldehyde, gently removed from the plastic by scraping and acquired on a BD FACSCanto II. Additionally, MDM or AM were plated onto Lab-Teks (Nunc) and infected at a known MOI of 1–10 bacteria per cell (~70% infectivity) in the presence of SAHA or vehicle control. After 3 h, Lab-Teks were washed with PBS and fixed with 2% paraformaldehyde prior to staining with rapid Auramine O staining set (Scientific Device Laboratory Inc) and Hoechst 33342 (Sigma-Aldrich). The numbers of bacteria per cell were counted using a fluorescent microscope (Olympus IX51) as described above.

### T Cell Co-culture Assays

MDM from IGRA positive individuals, infected with H37Ra in the presence of SAHA or DMSO, were washed after 24 h and co-cultured with thawed, CFSE-labelled (BioLegend; 0.5 μM, according to the manufacturer's protocol) autologous PBMC (2.5 × 10^6^ cells/ml; 10:1 ratio of PBMC to MDM). AM were co-cultured with allogeneic CFSE-labelled PBMC from a BCG-vaccinated, IGRA negative healthy control donor (2.5 × 10^6^ cells/well; 10:1 ratio of PBMC to AM). Half the volume of supernatant was removed and replaced with fresh medium at indicated timepoints. The concentrations of IFN-γ, GM-CSF, IL-10 (all BioLegend ELISA Max Deluxe kits), and TNF (Invitrogen ready-set-go kit) were quantified by ELISA, according to the manufacturer's protocol. On day 10 post co-culture, cells were Fc blocked (BioLegend Human TruStain FcX) and stained with fluorochrome-conjugated antibodies specific for CD4 (PerCP-Cy5.5), CD25 (APC; both eBioscience), CD3 (BV510), CD8 (APC-Fire750; both BioLegend). Cells were fixed and permeabilised for intranuclear staining (FoxP3 staining buffer set; eBiosciences) and stained with fluorochrome-conjugated antibodies specific for FoxP3 (PE; eBioscience). Cells were analysed on a BD FACS Canto II flow cytometer, as previously described (26). Alternatively, cells were restimulated with PMA (50 ng/ml) and ionomycin (500 ng/ml) in the presence of brefeldin A (5 μg/ml; all Sigma-Aldrich) for 5 h to assess intracellular cytokine production. PMA-stimulated cells were Fc blocked and stained with fluorochrome-conjugated antibodies specific for CD3 (BV510) and CD8 (APC-Fire750). Cells were fixed, permeabilised (Fix perm kit; Invitrogen) and stained with fluorochrome conjugated antibodies specific for GM-CSF (PE), IFN-γ (APC; both BioLegend), and TNF (PerCP-Cy5.5; eBioscience). Cells were acquired on the BD FACSCanto II. Cytokine production from CD3^+^ CD8^+^ and CD3^+^ CD8^−^ (CD4) CFSE^lo^ proliferating cells was analysed using FlowJo software as previously described (27).

### Colony Forming Units (CFU)

CFU were determined at day 0 (3 h post-infection), 3, 6, or 10, as indicated; cells were lysed with triton-X 100 (0.1%) and pooled with bacterial pellets (at all timepoints except day 0) from the centrifugation of supernatants. Bacteria were diluted in Middlebrook 7H9 broth and plated onto Middlebrook 7H10 agar supplemented with OADC (both Becton Dickinson) and cycloheximide (Sigma-Aldrich). CFU counts were performed 14- and 21-days post incubation at 37°C.

### Statistical Analysis

Statistical analyses were performed using GraphPad Prism 6 software. Statistically significant differences between two normally distributed groups were determined using Student's paired *t*-tests with two-tailed *P*-values. Differences between three or more groups were determined by one-way ANOVA with Tukey's multiple comparisons tests. Differences between two or more groups containing more than one variable were determined by two-way ANOVA with Sidak's multiple comparisons tests. *P* < 0.05 were considered statistically significant and denoted using an asterisk.

## Results

### SAHA Supports Aerobic Glycolysis and IL-1β Production in Human Macrophages Early in Response to Mtb

The metabolic function of human macrophages changes upon infection with Mtb toward increased utilisation of aerobic glycolysis (11). Mtb, however, perturbs host metabolism (28–30) to evade clearance by the innate immune response. Additionally people with increased risk of contracting TB, such as smokers, have an impaired ability to shift toward aerobic glycolysis in response to infection with Mtb (31). Therefore, drugs with the ability to promote glycolysis may be beneficial as treatment strategies for people with increased risk of TB.

The upregulation of macrophage effector functions, including changes in cellular metabolism, is governed by gene transcription and thus is under the control of HDAC. We, therefore, hypothesised that the HDAC inhibitor SAHA would modulate the metabolic function of human macrophages infected with Mtb. Emerging evidence suggests that SAHA modulates both pathogen and host epigenetics (32–36). Since HDAC inhibitors could potentially impact the deacetylation activity within Mtb, we first used iH37Rv to assess the impact of SAHA on the host independent of its effects on the bacteria.

**Figure 1:**
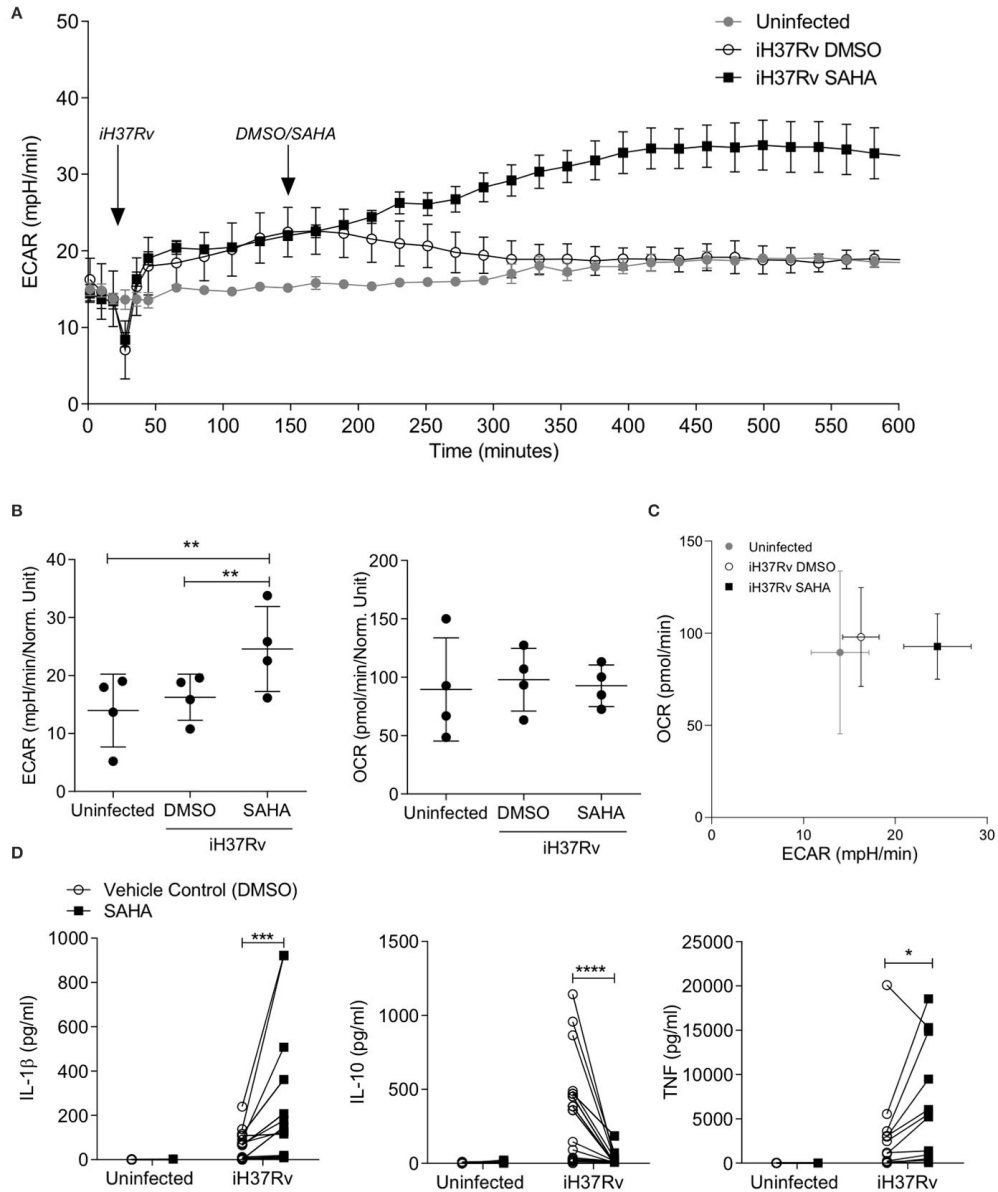
SAHA supports aerobic glycolysis and modulates cytokine production in human macrophages during early Mtb infection. Monocyte derived macrophages (MDM) were analysed on the Seahorse XFe24 Analyzer. The extracellular acidification rates (ECAR) and oxygen consumption rate (OCR) were recorded approximately every 20 min. After 30 min, the Seahorse Analyzer injected iH37Rv (MOI 1-10) into assigned wells. Two hours later, DMSO or SAHA were injected through the Seahorse Analyzer (at 150 min; as indicated by the arrows). The ECAR and OCR readings were then continually sampled in real time. **(A)** Representative time-course graph illustrating the ECAR of MDM in real-time response to stimulation with iH37Rv and treatment with SAHA or vehicle control (DMSO); 3 technical replicates ± SD. **(B)** Collated data (error bars indicate mean ± SD) from *n* = 4 independent experiments for ECAR and OCR at 500 min. **(C)** The phenogram illustrates the energetic profile of MDM by plotting ECAR vs. OCR at 500 min (±SD, *n* = 4). **(D)** MDM were stimulated with iH37Rv for 24 h and concentrations of IL-1β (*n* = 18), IL-10 (*n* = 18), and TNF (*n* = 12) present in the supernatants were quantified by ELISA. Each paired data point represents the average of technical replicates from a single donor treated with DMSO (empty circles) or SAHA (closed squares). Statistically significant differences between DMSO and SAHA were determined by one-way ANOVA with Tukey's multiple comparison test **(B)** or two-way ANOVA with Sidak's multiple comparison test **(D)**; *P < 0.05, **P < 0.01, ***P < 0.001, ****P < 0.0001.

We analysed the extracellular acidification rate (ECAR) and the oxygen consumption rate (OCR) of human monocyte derived macrophages (MDM) in response to stimulation with Mtb and treatment with SAHA (20 μM) in a Seahorse XFe24 Analyzer (Figure 1). Baseline measurements of ECAR and OCR, surrogates for glycolysis and oxidative phosphorylation, respectively, were recorded for 30 min prior to the addition of Mtb iH37Rv. MDM were then treated with SAHA or the vehicle control (DMSO) 120 min post the addition of iH37Rv. ECAR and OCR were tracked in real-time over a 10-h period. The glycolytic rate (ECAR) of human MDM increased 15 min post the addition of iH37Rv (at 45 min; Figure 1A). The addition of SAHA at 150 min augmented the increasing glycolytic rate between 200 and 400 min, whereas the ECAR of MDM that received vehicle control returned to the baseline rate of the uninfected, untreated control (Figure 1A). Collated data from *n* = 4 independent experiments demonstrates that, at 500 min, SAHA significantly increased the ECAR (glycolysis) of human MDM stimulated with Mtb compared with DMSO-treated controls and uninfected MDM (Figure 1B, left; *P* < 0.01). Conversely, the OCR (oxidative phosphorylation) was not affected by the addition of SAHA (Figure 1B, right). A phenogram illustrating ECAR vs. OCR at 500 min shows the metabolic state of MDM infected in the presence of SAHA or vehicle control compared with uninfected cells (Figure 1C). The addition of SAHA in the absence of infection did not alter the metabolic profile of human MDM (data not shown). Twenty-four hours post stimulation with iH37Rv, SAHA-treated macrophages exhibited significantly reduced ECAR with no effect on OCR (Supplemental Figure 1). These data indicate that SAHA supports the rapid shift to glycolysis in human macrophages during the early response to Mtb infection.

Increased glycolysis in proinflammatory murine M1-type macrophages is associated with increased production of mature IL-1β but does not affect TNF production (37), whereas increased oxidative phosphorylation in homeostatic M2-type macrophages is linked to IL-10 production (38). In addition, IL-1β production and increased aerobic glycolysis promotes early clearance of Mtb from human and murine macrophages (11, 29). We assessed the effect of SAHA at a range of concentrations (from 5 to 40 μM) on the production of IL-1β and IL-10 in human MDM stimulated with iH37Rv (Supplemental Figure 2A). SAHA at 20 and 40 μM optimally induced IL-1β production whereas SAHA abrogated IL-10 at all concentrations examined. Since IL-1β release can result from pyroptosis (39), we analysed the effect of SAHA (20 μM) on human MDM cell death (Supplemental Figure 2B). MDM were treated with SAHA or vehicle control and stimulated with iH37Rv, infected with H37Ra or treated with cycloheximide as a positive control for cell death. After 24 h, MDM were stained with propidium iodide and Hoechst; cell death was analysed. SAHA- and DMSO-treated MDM were washed and cultured in RPMI containing 10% FBS for the remaining time-points. SAHA was therefore used at a concentration of 20 μM which did not significantly promote cell death apart from in cycloheximide-treated MDM on day 3 (*P* < 0.05) when compared with control (Supplemental Figure 2B).

MDM from healthy control donors were stimulated with iH37Rv in the presence of SAHA (20 μM) or vehicle control. Since IL-1β and TNF are fundamental during the early host response to Mtb (11, 40–42) whereas IL-10 promotes TB disease progression in mice (43) and interrupts host defence in humans (44), we determined the concentrations of IL1-β, IL-10, and TNF by ELISA 24 h post stimulation (Figure 1D). SAHA significantly increased the production of IL-1β (*P* < 0.001) and TNF (*P* < 0.05) in human MDM stimulated with iH37Rv (Figure 1D). Conversely, SAHA significantly decreased production of IL-10 (*P* < 0.0001; Figure 1D).

To ensure that the increased glycolytic rate and increased IL-1β production were not a result of increased bacterial load inside SAHA-treated macrophages, we examined the effect of SAHA on phagocytosis in human MDM and AM (Supplemental Figures 2C–E). SAHA did not significantly alter the uptake of latex beads or Mtb.

Collectively, these data indicate that SAHA significantly increased glycolysis early in the response to stimulation with Mtb in human macrophages. Furthermore, SAHA significantly increased IL-1β and TNF production and significantly reduced IL-10, compared with vehicle control, in human macrophages stimulated with iH37Rv. These effects were not associated with increased cell death or increased bacterial load.

### SAHA Modulates Cytokine Production in the Context of Live Mtb Infection in Human MDM and Alveolar Macrophages

**Figure 2:**
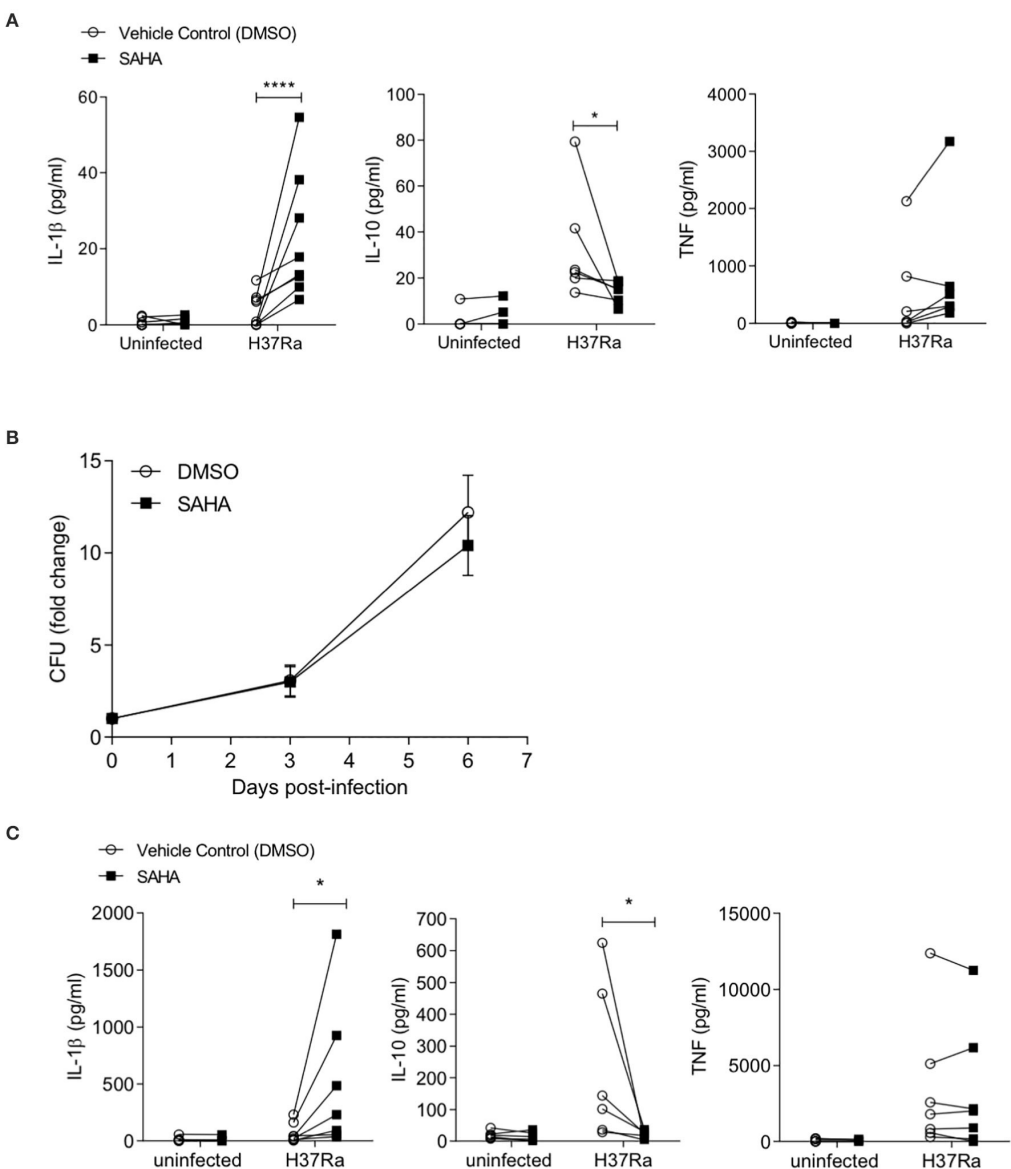
SAHA modulates cytokine production in the context of live Mtb H37Ra infection in human healthy control MDM and alveolar macrophages. **(A)** MDM were infected with Mtb H37Ra (MOI 1-10) in the presence of SAHA or vehicle control (DMSO) for 24 h. The concentrations of IL-1β (*n* = 8), IL-10 (*n* = 6), and TNF (*n* = 8) present in the supernatants were quantified by ELISA. **(B)** MDM infected with Mtb H37Ra (MOI 1-10) were lysed on day 0 (3 h post-infection), day 3, and day 6. CFU were enumerated on Middlebrook 7H10 agar supplemented with OADC on day 21 post lysis. CFU are presented as fold change from day 0 and data points represent the average ± SD of *n* = 3 independent experiments. **(C)** Human AM (*n* = 7) were infected with H37Ra (MOI 1-10) in the presence of SAHA or DMSO. After 24 h, the concentrations of IL-1β, IL-10, and TNF present in the supernatants were measured by ELISA. Each paired data point represents the average of technical replicates from a single donor treated with DMSO (empty circles) or SAHA (closed squares). Statistically significant differences between DMSO and SAHA were determined by two-way ANOVA with Sidak's multiple comparison test **(A,C)** or by paired Student's *t*-test **(B)**; *P < 0.05, ****P < 0.0001.

To ensure this phenotype was recapitulated in the context of a live infection, we infected human MDM from healthy control donors with Mtb H37Ra in the presence of SAHA or vehicle control. After 24 h, the concentrations of IL-1β, IL-10, and TNF were quantified by ELISA (Figure 2A). Although the overall magnitude of the cytokine response was reduced in MDM infected with live H37Ra (Figure 2A) compared with iH37Rv (Figure 1D), SAHA significantly increased IL-1β production (*P* < 0.0001) and significantly reduced IL-10 production (*P* < 0.05) without affecting TNF production. Cells were lysed on day 0 (3 h post-infection), day 3, and day 6, and CFU were enumerated on Middlebrook agar supplemented with OADC (Figure 2B). SAHA did not significantly affect bacterial killing in human MDM.

The alveolar macrophage is the first cell to encounter Mtb and is thought to be ineffective at killing the bacteria (45–47). Therefore, the AM is a target cell for potential inhalable adjunctive therapies aimed at boosting immune function. We isolated human alveolar macrophages from BALF and infected them with Mtb in the presence of SAHA or vehicle control (Figure 2C). We found that HDAC inhibition with SAHA significantly increased IL-1β production (*P* < 0.05) and significantly reduced IL-10 production (*P* < 0.05) without altering TNF production in human alveolar macrophages infected with Mtb. Five out of seven of the AM donors were smokers; when we excluded non-smokers from the analysis SAHA maintained the ability to significantly boost IL-1β (*P* < 0.05; data not shown).

### Treating MDM With SAHA Enhances Downstream Effector T Cell Responses to Mtb

**Figure 3:**
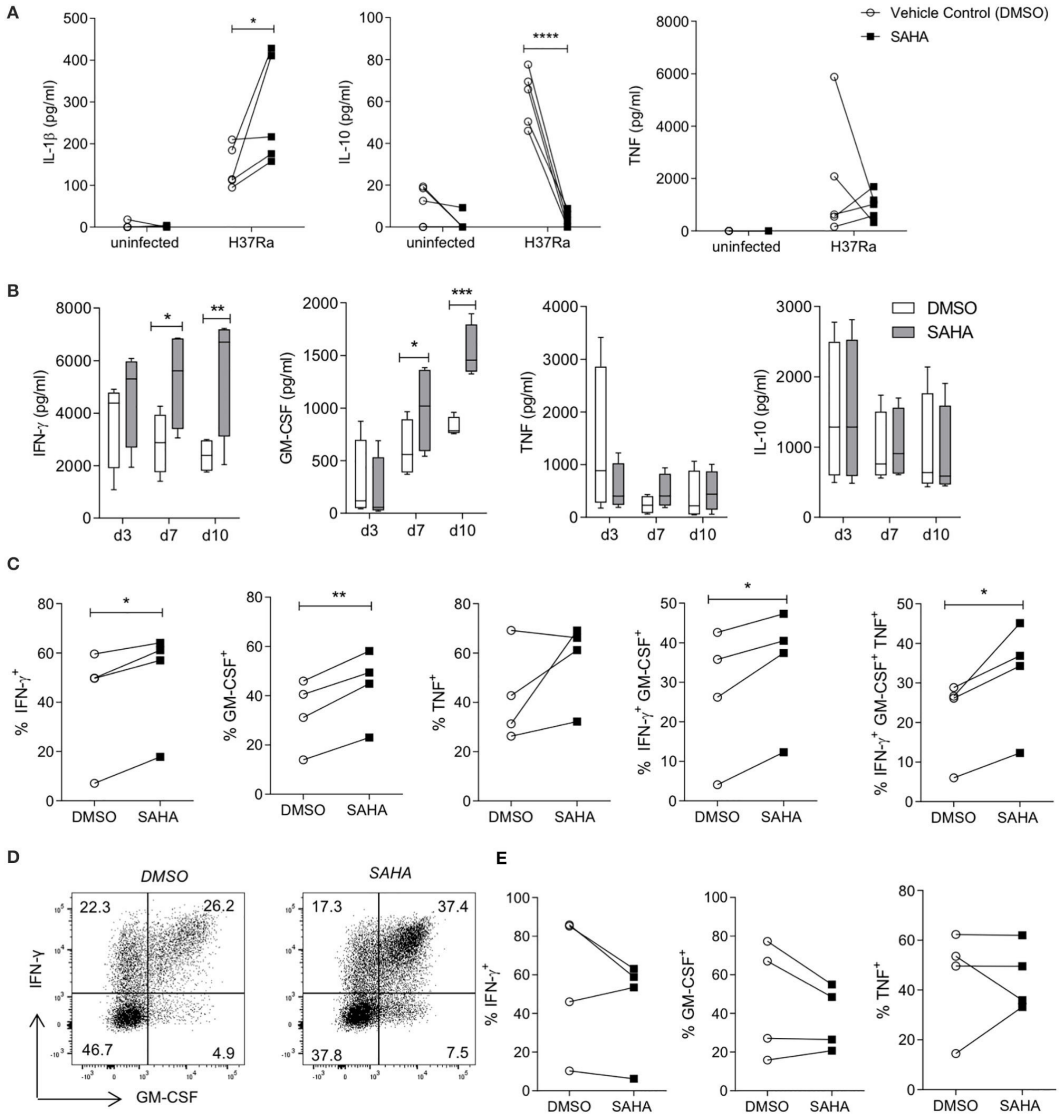
SAHA-treated MDM enhanced downstream effector CD4 T cell responses to Mtb. MDM differentiated from the PBMC of IGRA positive individuals were infected with H37Ra (MOI 1-10) in the presence of SAHA or vehicle control (DMSO). **(A)** After 24 h, the concentrations of IL-1β, IL-10 and TNF were quantified by ELISA (*n* = 5). MDM were washed and co-cultured with CFSE-labelled PBMC from autologous donors. **(B)** The concentrations of IFN-γ, GM-CSF, TNF, and IL-10 present in the co-cultured supernatants on the indicated days were analysed by ELISA; collated data from *n* = 4 experiments, error bars indicate ± SD. **(C–E)** On day 10 post co-culture, PBMC were removed, stimulated with PMA/ionomycin in the presence of brefeldin A, or left unstimulated. Cells were stained with fluorochrome-conjugated antibodies specific for CD3, CD8, IFN-γ, TNF and GM-CSF, and analysed by flow cytometry. **(C)** Graphs illustrate collated data for the frequencies of cells producing cytokines in the population of CD3^+^CD8^−^ (CD4^+^) T helper cells that are proliferating (CFSE^lo^) in response to stimulation with Mtb infected macrophages (*n* = 4). Cells were gated on the basis of forward and side scatter, doublets were then excluded and the population of proliferating CFSE^lo^ cells were gated. The Th cell subpopulation was gated within proliferating cells, then cytokines were examined within these populations. **(D)** Representative dot plots illustrate co-staining of GM-CSF with IFN-γ from proliferating CD4 (CD3^+^ CD8^−^) T cells. **(E)** Cytokine production from proliferating CD8^+^ T cells was assessed. Statistically significant differences between DSMO and SAHA treated groups were determined by two-way ANOVA with Sidak's multiple comparisons test **(A,B)** or paired *t*-test **(C,E)**; ^*^P < 0.05, **P < 0.01, ***P < 0.001.

Infiltrating monocytes that are recruited to the lung during infection and then become MDM are critical to controlling and killing Mtb (47). Moreover, both MDM and tissue resident AM are crucial for reactivating infiltrating effector T cells in the tissue. We utilised blood from interferon gamma release assay (IGRA) positive individuals to allow us to determine if treating Mtb-infected macrophages with SAHA had an effect on T cell responses. First we analysed the effect of SAHA on MDM from IGRA-positive donors (Figure 3A). HDAC inhibition with SAHA in infected MDM from IGRA positive donors significantly promoted IL-1β (*P* < 0.05) and reduced IL-10 (*P* < 0.0001) and did not significantly affect TNF production. We then established a co-culture assay in order to assess the impact of the increased glycolysis and IL-1β production and concomitantly reduced IL-10 production in infected macrophages treated with SAHA on the downstream T cell response.

T cells can be both protective and pathogenic during Mtb infection depending on their function and the stage of TB disease (48). IFN-γ and GM-CSF have emerged as critical T cell cytokines in the control of Mtb and have been shown to have additive effects on macrophage-mediated killing of Mtb (49). Therefore, we assessed if SAHA-treated macrophages could modulate T cell production of IFN-γ and GM-CSF.

MDM from IGRA-positive donors were infected with Mtb H37Ra in the presence of SAHA or vehicle control. After 24 h, MDM were thoroughly washed to remove SAHA/DMSO and co-cultured with autologous CFSE-labelled PBMC. The concentrations of IFN-γ, GM-CSF, IL-10, and TNF were quantified by ELISA on day 3, 7, and 10 post co-culture (Figure 3B). PBMC co-cultures containing SAHA-treated MDM infected with Mtb exhibited significantly increased concentrations of IFN-γ and GM-CSF at day 7 (*P* < 0.05) and day 10 (*P* < 0.01, *P* < 0.001, respectively) compared with PBMC co-cultured with DMSO-treated, Mtb-infected MDM. No differences were observed in the production of TNF or IL-10 (Figure 3B).

In order to determine which cells in the co-culture system were producing these cytokines, we used flow cytometry to assess cytokine production in subpopulations of the proliferating (CFSE^lo^) cells. Representative histograms illustrate the frequencies of proliferating cells (CFSE^lo^) in response to stimulation by uninfected autologous MDM, or MDM infected with Mtb in the presence of SAHA or DMSO (Supplemental Figure 3A). The frequencies of proliferating PBMC stimulated by Mtb-infected MDM treated with SAHA were not significantly altered compared with DMSO (Supplemental Figure 3A; collated data, right). Furthermore, over 90% of proliferating cells were CD3^+^ T cells, and this was not altered by treating the MDM with SAHA (Supplemental Figure 3B). In addition, SAHA did not significantly alter the frequencies of CD4^+^ T helper (Th) cells or CD8^+^ cytotoxic T (Tc) cells compared with DMSO (Supplemental Figure 3C).

PBMC were restimulated with PMA and ionomycin in the presence of brefeldin A to assess cytokine production from proliferating T cell populations. Proliferating CD4 Th cells (gated on CD3^+^ CD8^−^ cells as PMA stimulation reduces CD4 surface expression), stimulated by autologous SAHA-treated MDM infected with Mtb, exhibited significantly increased frequencies of IFN-γ^+^ (*P* < 0.05), GM-CSF^+^ (*P* < 0.01), IFN-γ^+^ GM-CSF^+^ (*P* < 0.05), and IFN-γ^+^ GM-CSF^+^ TNF^+^ (*P* < 0.05) cells (Figure 3C). Representative dot plots illustrate the co-staining of IFN-γ and GM-CSF (Figure 3D). No significant differences were observed in cytokine production within proliferating CD8^+^ Tc cells stimulated by autologous DMSO/SAHA-treated MDM infected with Mtb (Figure 3E).

These results indicate that treating human macrophages from IGRA-positive donors with SAHA promoted downstream effector Th cell responses.

### SAHA Treated AM Enhance BCG-Primed CD4 Th Cell Responses to Mtb Infection

Having established that treating Mtb-infected MDM with SAHA resulted in increased effector Th cell function, we next sought to determine whether SAHA-treated AM infected with Mtb would promote effector T cell responses in healthy control individuals (IGRA negative) who had been BCG vaccinated and are therefore able to respond to Mtb antigens (PPD) *in vitro*. Due to clinical and ethical restrictions, AM and PBMC were isolated from different donors, therefore the allogeneic response was controlled for throughout the experiment.

AM were infected with Mtb H37Ra in the presence of SAHA or vehicle control. After 24 h, AM were thoroughly washed to remove SAHA/DMSO and co-cultured with CFSE-labelled PBMC from a BCG-vaccinated donor (IGRA negative but responds to PPD antigens *in vitro*). Uninfected AM co-cultured with PBMC (background allogeneic response) and AM infected with Mtb H37Ra and not co-cultured with PBMC were assayed as controls.

**Figure 4:**
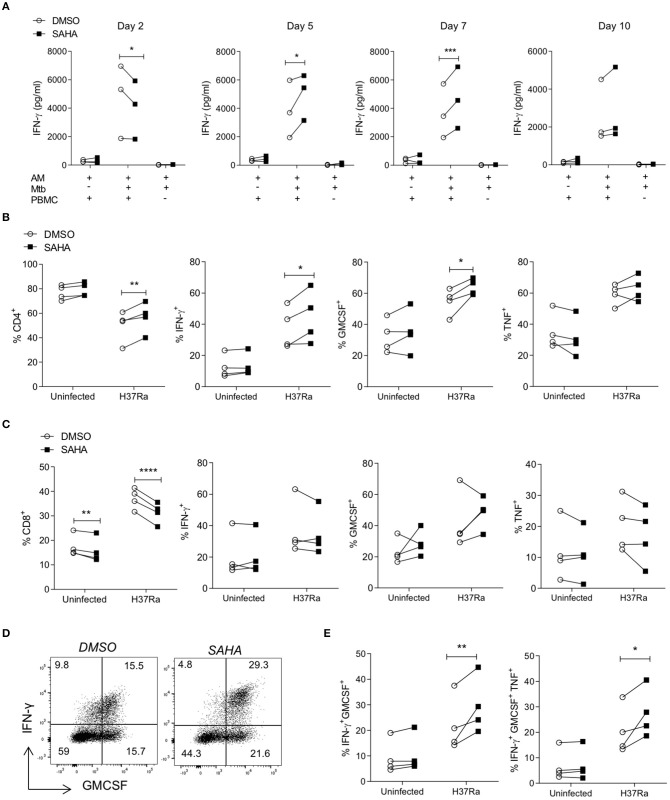
SAHA treated AM enhanced BCG-primed CD4 Th cell responses to Mtb infection. Human AM were infected with H37Ra (MOI 1-10) in the presence of SAHA or DMSO. After 24 h, AM were washed and co-cultured with CFSE-labelled PBMC from a BCG-vaccinated healthy donor (IGRA negative) who responds to PPD antigens *in vitro*. Uninfected AM co-cultured with PBMC and Mtb-infected AM not co-cultured with PBMC were assayed in parallel as controls. **(A)** The concentrations of IFN-γ present in the supernatants on the indicated days were analysed by ELISA (*n* = 3). On day 10 post co-culture, PBMC were removed, stimulated with PMA/ionomycin in the presence of brefeldin A, or left unstimulated. **(B,C)** Cells were stained with fluorochrome-conjugated antibodies specific for CD3, CD4, CD8, IFN-γ, TNF and GM-CSF, and analysed by flow cytometry. Cells were gated on the basis of forward and side scatter, doublets were then excluded and the population of proliferating CFSE^lo^ cells were gated. The CD4 and CD8 cell subpopulations were gated within proliferating cells, then cytokines were examined within these populations. Graphs illustrated collated data (*n* = 4) from unstimulated samples for the frequencies of proliferating cells expressing CD4 (**B**; left) or CD8 (**C**; left) and cytokine production from stimulated samples within the population of proliferating (CFSE^lo^) CD3^+^ CD8^−^ (CD4^+^) T helper cells **(B)** and CD3^+^ CD8^+^ cytotoxic T cells **(C)**. **(D)** Representative dot plots illustrate co-staining of IFN-γ and GM-CSF within the CFSE^lo^ CD3^+^ CD8^−^ Th cell gate. **(E)** Collated data shows the frequencies of IFN-γ^+^ GM-CSF^+^ double-positive and IFN-γ^+^ GM-CSF^+^ TNF^+^ triple-positive cells in the CD4 Th cell population that are proliferating. Statistically significant differences between DSMO and SAHA treated groups were determined by two-way ANOVA with Sidak's multiple comparisons test; *P < 0.05, **P < 0.01, ***P < 0.001, ****P < 0.0001.

The concentrations of IFN-γ produced on day 2, day 5, day 7, and day 10 post co-culture were quantified by ELISA (Figure 4A). Notably, allogeneic PBMC co-cultured with Mtb-infected AM produced increased IFN-γ compared with background allogeneic responses or AM cultured alone. On day 2, PBMC stimulated with Mtb-infected, SAHA-treated AM exhibited significantly reduced IFN-γ production compared with DMSO control (*P* < 0.05). However, on day 5 and day 7, PBMC stimulated with Mtb-infected, SAHA-treated AM exhibited significantly increased IFN-γ production compared with PBMC stimulated with DMSO-treated, Mtb-infected AM (*P* < 0.05 and *P* < 0.001, respectively). By day 10, there was no significant difference in IFN-γ production. The concentrations of GM-CSF, TNF, and IL-10 present in the supernatant on day 5 are not significantly altered by treating the AM with SAHA compared with control (Supplemental Figure 4A).

CFSE^lo^ proliferating PBMC were analysed by flow cytometry on day 10, as above (section Treating MDM with SAHA Enhances Downstream Effector T Cell Responses to Mtb). Treating infected AM with SAHA significantly increased the frequencies of CD4^+^ proliferating T cells (Figure 4B; *P* < 0.01) and significantly reduced the frequencies of proliferating CD8^+^ T cells (Figure 4C; *P* < 0.0001). Proliferating CD4^+^ Th cells activated by SAHA-treated, infected AM exhibited significantly increased frequencies of cells producing IFN-γ (*P* < 0.05) and GM-CSF (*P* < 0.05) compared with control (Figure 4B). Treating the AM with SAHA did not significantly impact the ability of CD4^+^ Th cells to produce TNF. In addition, treating the AM with SAHA had no impact on the ability of CD8^+^ Tc to produce cytokine (Figure 4C). Representative dot plots show the co-staining of IFN-γ and GM-CSF in proliferating CD4 T cells (Figure 4D). Collated data indicates that SAHA-treated, Mtb infected AM promote significantly higher frequencies of IFN-γ^+^ GM-CSF^+^ double-positive (*P* < 0.01) and IFN-γ^+^ GM-CSF^+^ TNF^+^ triple-positive (*P* < 0.05) CD4^+^ Th cells compared with control (Figure 4E).

Our group have previously shown that the lung environment and specifically AM can promote the induction of Treg cells (23). Taken together with the data demonstrating that SAHA abrogated IL-10 production from AM, and since IL-10 is a major driver of Treg induction, we analysed the frequencies of Treg cells present in the co-culture. Representative plots showing the gating strategy for Treg cells and collated data indicate that treating infected AM with SAHA did not significantly alter the frequencies of CD4^+^ Treg cells compared with control (Supplemental Figures 4B,C).

The autologous MDM-PBMC co-culture (of BCG vaccinated, IGRA negative donor who positively responds to PPD antigens *in vitro*) was assayed (Supplemental Figures 4D–F) as a control for the allogeneic AM-PBMC co-culture and also to assess if the findings could be recapitulated in the context of IGRA negative donors. The concentrations of IFN-γ present in the supernatants were analysed (Supplemental Figure 4D). Similar to the PBMC co-cultured with allogeneic AM and the IGRA-positive autologous model, IGRA negative PBMC stimulated with SAHA-treated, Mtb-infected autologous MDM exhibited significantly reduced IFN-γ production at day 3 (*P* < 0.0001) however; by day 7 and 10, PBMC secreted significantly more IFN-γ compared with controls (*P* < 0.0001; Supplemental Figure 4D). Dot plots show increased frequencies of Th cells co-producing IFN-γ and GM-CSF (Supplemental Figure 4E). CFU were enumerated at on day 0, day 3, and day 10 (Supplemental Figure 4F). MDM treated with SAHA exhibit reduced CFU at day 10 compared with control. MDM co-cultured with PBMC exhibit profoundly reduced CFU compared to MDM alone, as expected, but without a substantial difference between the treatment groups.

These data demonstrate that in the context of lung resident AM from healthy donors, SAHA can promote downstream Th cell responses, similar to the effects observed in the cells isolated from IGRA-positive individuals.

Taken together, these data illustrate that SAHA modulates metabolic function and affects the cytokine profile of macrophages infected with Mtb, and this results in enhanced Th cell effector function.

## Discussion

The metabolic function of macrophages is emerging in the literature as a gatekeeping node in mediating the immune response to danger signals. Moreover, perturbance of macrophage metabolic function by Mtb or other environmental cues is associated with increased risk of TB disease and enhanced pathogenicity of the bacteria (24, 28, 29). Therefore, modulating the metabolic function of macrophages will be key to translating the burgeoning field of immunometabolism toward clinical benefit for patients.

Our hypothesis-driven human research aimed to provide proof-of-concept data for repurposing the FDA approved HDACi, SAHA, as an immune-augmenting therapy. These data can be used to inform further experimental models of infection *in vivo*.

Our data indicates that the use of the HDACi, SAHA, on human macrophages modulates both innate and adaptive immune function in response to infection with Mtb. Treating macrophages with SAHA resulted in significantly increased glycolysis early in the response to Mtb infection. Importantly this effect could be solely attributed to host macrophage responses to HDACi since iH37Rv was used to ensure direct effects of SAHA on the bacteria did not confound the experiment. HDACi have been reported to increase mitochondrial reactive oxygen species (ROS) in human macrophages infected with *S. Typhimurium* or *E. coli* (22). This is in keeping with our observation that SAHA promoted the early shift to glycolysis since mitochondrial ROS is associated with increased glycolysis in macrophages (50). The evidence for this increased glycolysis was further supported by the macrophage shift toward a more proinflammatory phenotype; producing more IL-1β and less IL-10. This increased glycolysis coupled with increased IL-1β is a well-documented phenotype in activated macrophages (37) and has previously been shown by our group in the context of Mtb infection (11, 29, 51). Inhibiting glycolysis *in vitro* has been reported to have no effect on TNF production from macrophages (11, 37). Infecting MDM with H37Ra in the presence of SAHA did not affect TNF production, in keeping with published literature that demonstrates the TNF is not associated with the shift to glycolysi*s in vitro*. However, we also report here that treating human MDM with SAHA not only promoted glycolysis and IL-1β but also promoted TNF production in response to stimulation with iH37Rv. This indicates that TNF may be directly or indirectly linked to increased glycolysis in the context of iH37Rv stimulation in human macrophages. Work recently published by our group supports this data by showing that promoting glycolysis using an iron chelator also increased TNF production in human macrophages stimulated with iH37Rv (51). Furthermore, inhibition of glycolysis using 2-deoxyglucose in an *in vivo* mouse model of Mtb infection resulted in decreased *ex vivo* TNF production from interstitial macrophages from the lung (47). These data collectively suggest that the relationship between the metabolic function of macrophages and their ability to produce TNF is complex and therefore warrants further study.

We and others have previously shown that IL-1β (11, 40, 41) and TNF (41, 42) are critical for control of Mtb, whereas IL-10 inhibits bacterial clearance (44). The observed increase in IL-1β production and concomitant decrease in IL-10 may shift the immune response in favour of the host. Although SAHA promoted TNF production in the context of iH37Rv stimulation, it did not increase TNF production in the context of infection with H37Ra. This suggests that whilst the net proinflammatory effect is greater in the context of iH37Rv (with both IL-1β and TNF increased in response to SAHA), there is still a shift toward increased proinflammatory responses in the live infection (increased IL-1β, unchanged TNF, and decreased anti-inflammatory IL-10). Interestingly, SAHA promoted IL-1β production in murine bone marrow derived macrophages and human dendritic cells stimulated with LPS (52), suggesting SAHA may have wider applications as a modulator of macrophage function in other settings of infectious disease and cancer.

AM exhibit reduced capacity to shift toward aerobic glycolysis (47), which is accentuated in smokers, who are at increased risk of TB (31). In addition, emerging evidence suggests that multidrug-resistant Mtb does not induce a shift toward glycolysis in macrophages (28). Therefore, drugs with the ability to promote this shift toward utilising glycolysis, independent of the bacteria, may have significant clinical promise.

In the context of liver cancer, SAHA has been shown to inhibit HIF-1α expression and nuclear translocation (53, 54). Because the shift toward utilising aerobic glycolysis in proinflammatory macrophages is governed by HIF-1α stabilisation (55), this finding in cancer may appear incongruous to our findings that SAHA promoted the shift to glycolysis. These disparities may, at least in part, be due to timing; although SAHA promoted glycolysis early in response to stimulation with iH37Rv, it reduced glycolysis at the later timepoint of 24 h.

The activation status, cytokine production, and metabolic function of APC can influence T cell activation (56), therefore, we analysed the effect of treating Mtb-infected macrophages with SAHA on downstream T cell responses. We found significantly increased IFN-γ and GM-CSF production. Importantly, the flow cytometry data demonstrates that the responding Th cells were able to produce IFN-γ, GM-CSF, and TNF concurrently. Increased IFN-γ and GM-CSF production is thought to be protective during TB disease (57, 58) and these cytokines exhibit an additive effect in promoting macrophage killing of Mtb *in vivo* (49). Moreover, cytokine polyfunctionality is thought to be beneficial over monofunctional T cell responses (59).

Although SAHA completely abrogated the production of IL-10 in macrophages infected with Mtb, the co-culture of macrophages with PBMC is IL-10 replete; indicating that the early abrogation of IL-10 production in macrophages is later rescued by subsequent responding T cells. This is important in order to ensure that these increased early inflammatory responses are balanced to limit excessive inflammation and damage to the lung (60).

Since IL-10 is a well-established suppressor of T cell activation (61), the ability of SAHA to reduce IL-10 production in infected macrophages likely contributes toward the enhanced T cell responses observed. In addition, IL-1β has been shown to drive a polyfunctional non-classical Th1-type response in the context of *Listeria monocytogenes* infection (62). We have previously shown that increased IFN-γ^+^ GM-CS^+^ TNF^+^ polyfunctional cells are associated with the non-classical Th1 cell phenotype (27), therefore, the increased IL-1β in SAHA-treated Mtb-infected macrophages may be directly driving the enhanced polyfunctional Th1-type response. It is also plausible that the reduced ECAR observed in infected SAHA-treated macrophages after 24 h may promote better antigen presentation and T cell responses, consistent with published observations in dendritic cells whereby an initial increase in glycolysis is required for activation but subsequently reduced glucose uptake increases bioavailability of glucose for T cell activation (56). Thus, the metabolic phenotype induced by SAHA may be beneficial *in vivo* to support early clearance events by macrophages and then subsequently promote T helper cell responses.

The recapitulation of these findings in AM from healthy donors and T cells from a BCG-vaccinated healthy donor suggests that SAHA may have potential as a vaccine adjuvant delivered directly to the lungs. Additionally, because of SAHA's ability to promote IL-1β in AM from smokers, this approach may be useful in contexts where sessile, metabolically exhausted AM fail to support early clearance (31). BCG vaccination can induce trained immunity which confers non-specific protection against unrelated infections (63). The mechanisms underpinning trained immunity are changes in epigenetics and metabolic function (13, 14). Therefore, we hypothesise that SAHA may be able to modulate the “trained” phenotype in macrophages which may in turn effect the subsequent T cell response.

SAHA is directly cytotoxic to T cells in the context of cutaneous T cell lymphoma (64). Our data, however, indicates that SAHA can indirectly promote T helper cell function downstream of human macrophages in the context of Mtb infection. The role of the T cell in mediating protection vs. pathology in TB is debatable (65–70). We hypothesise that targeted delivery of SAHA to the lung may be beneficial; firstly to promote macrophage-mediated immunity to Mtb and secondly to refresh the population of T cells present in the lung during the disease state. Newly infiltrating T cells will therefore be reactivated *in situ* by SAHA-treated macrophages, that can enhance T helper cell effector functions.

Research around host-directed therapies have focused on anti-inflammatories to limit pathology in established TB disease, however, there is also a role for cell targeted immune-augmentation (51, 71). This strategy might have a role in early infection events, or to improve myeloid killing of drug resistant bacilli (28). We recently demonstrated *in vivo* efficacy of this approach, using inhaled macrophage homing micro-particles that contain all-trans retinoic acid which drives macrophage anti-TB responses (72, 73). Targeting HDACs has previously been postulated to have potential as an adjunct host-directed therapy for TB (10, 74). Our data is also supported by published *in vivo* data showing that Tubastatin A, a selective HDAC6 inhibitor, reduced IL-10, and enhanced Th1 responses in mice infected with Mtb H37Ra (9). Based on the current cellular study and on recently published data indicating that HDACi enhanced anti-mycobacterial responses in human macrophages (10), SAHA warrants further investigation as an immunosupportive agent.

### Study Limitations

Although treating human macrophages with SAHA showed promising effects on innate and adaptive immune responses to Mtb, our study ambiguously did not show any significant effects on bacterial load. SAHA has previously been shown to have no direct effect on growth of Mtb in an *in vitro* axenic culture model, however, it elicited a modest reduction in bacterial burden in THP1 macrophages co-treated with SAHA and rifampicin compared to rifampicin alone (35). Considering this and our current study, we postulate that combining SAHA with conventional antibiotic therapies may aid killing of the pathogen through innate and adaptive mechanisms. Moreover, HDACi including SAHA have been shown to prevent ototoxic hearing loss (75) caused by aminoglycoside antibiotics and may therefore have beneficial off-target affects.

Our data indicates that SAHA augments the innate immune response to Mtb via increased glycolysis. This boosted innate immune response propagates enhanced polyfunctional Th cell responses *in vitro*. Although SAHA may have therapeutic potential in settings where the immune response is impaired, we also recognise that such an approach may be detrimental during active TB disease.

The effects observed on T cells stimulated with Mtb-infected AM are confounded by the allogeneic nature of this co-culture. Although the T cell response is increased over the background allogeneic response, the differences in Th cell function may be due to a bystander effect. Whilst we cannot fully rule this out, we have observed that SAHA-treated AM stimulated with LPS exhibit increased IL-1β and decreased IL-10 production and when these AM are co-cultured with PBMC, they do not elicit a T cell response over the background allogeneic response and no differences were observed between the treatment groups (data not shown).

We used an avirulent Mtb strain to model the successful host immune response, though we have previously shown that this is a good model for the viable virulent pathogen in these ex-vivo experiments (11, 29). Additionally, the use of irradiated H37Rv allows us to analyse the effects of SAHA on the host without the confounding factor of the drug manipulating mycobacterial epigenetics and causing an effect.

Whilst it is likely that SAHA induces epigenetic changes via HDAC inhibition, the current study cannot rule out the possibility of non-specific hyperacetylation of other proteins, as HDAC also deacetylate non-histone proteins (76).

### Conclusion

Treating human macrophages with SAHA enhanced proinflammatory function by promoting the early shift to glycolysis. In turn, these SAHA-treated, Mtb infected macrophages enhanced the T helper cell response downstream compared with vehicle control-treated infected macrophages.

Our data provides a proof-of-concept in primary human macrophages and T cells to advance SAHA toward pre-clinical *in vivo* studies. It also provides new promising rationale for targeting epigenetics to modulate human immunometabolic processes in macrophages.

## Data Availability Statement

The datasets generated for this study are available on request to the corresponding author.

## Ethics Statement

The studies involving human participants were reviewed and approved by St. James's Hospital and Tallaght University Hospital Research Ethics Committee. The patients/participants provided their written informed consent to participate in this study.

## Author Contributions

SB, DC, JK, and PD were responsible for the experimental conception, design, and analysis. SB, DC, AC, CÓ, KG, and JP carried out the experimental work. SB co-wrote the manuscript with DC and it was revised by all authors. JK was responsible for obtaining clinical samples. All authors contributed to the article and approved the submitted version.

## Conflict of Interest

The authors declare that the research was conducted in the absence of any commercial or financial relationships that could be construed as a potential conflict of interest.
