# Supplementary material for: Inhibiting Histone Deacetylases in Human Macrophages Promotes Glycolysis, IL-1β, and T Helper Cell Responses to Mycobacterium tuberculosis
Source: Front Immunol. 2020 Jul 23;11:1609. doi: 10.3389/fimmu.2020.01609 (PMC7390906; doi:10.3389/fimmu.2020.01609)
Supplement: Supplementary file 1 [file Data_Sheet_1.docx]

Supplementary Figures

**Supplemental Figure 1: SAHA reduced the glycolytic rate of human MDM 24 hours post-infection.** PBMC were isolated from healthy control buffy coats and MDM were adherence purified. MDM were gently scraped and seeded onto Seahorse plates. Cells were stimulated with iH37Rv in the presence of SAHA or vehicle control (DMSO) and analysed on the Seahorse XFe24 Analyzer 24 hours later. The extracellular acidification rate (ECAR) and oxygen consumption rate (OCR) were recorded. Each paired data point represents an individual donor (n=7). Statistically significant differences between DSMO and SAHA treated groups were determined by Student’s paired T-tests; * *P*<0.05.

**Supplemental Figure 2: SAHA at a concentration of 20 µM optimally increased IL-1β production and decreased IL-10 without affecting viability or uptake.** PBMC were isolated from healthy control buffy coats and MDM were adherence purified. MDM (n=3) were infected with iH37Rv in the presence of increasing concentrations of SAHA (5, 10, 20 and 40 µM) or corresponding volumes of vehicle control (DMSO). Uninfected MDM were assayed in parallel as a control. After 24 hours the concentrations of IL-1β and IL-10 were quantified by ELISA (A; error bars indicate mean ±SD). MDM (n=4) were infected with H37Ra or stimulated with iH37Rv or treated with cycloheximide as a positive control for cell death, in the presence of SAHA (20 μM) or vehicle control. Cells were stained with propidium iodide and Hoechst. Cell death was quantified at the indicated time-points on the Cytell Cell Imaging system (B; error bars indicate mean ±SD). (C) MDM were treated with DMSO or SAHA and cultured with fluorescently labelled latex beads, washed and analysed by flow cytometry (n=5). Human MDM (D) or AM (E) were treated with DMSO or SAHA and infected with H37Ra. The MOI was determined by Auramine O staining; graphs show representative donors, error bars represent mean ±SD. Statistically significant differences between DSMO- and SAHA-treated groups were determined by two-way ANOVA with Bonferroni’s multiple comparisons test (A&B), Student’s t-test (C-E); * *P*<0.05, NS=not significant.

**Supplemental Figure 3: Gating strategy and collated data showing that treating MDM from IGRA-positive donors with SAHA does not significantly affect the proliferation or the frequencies of responding CD3^+^ T cells.** PBMC were isolated from the venous blood of consenting IGRA positive donors. MDM were adherence purified and autologous PBMC were cryopreserved. MDM were infected with H37Ra for 24 hours in the presence of SAHA or vehicle control, thoroughly washed and co-cultured with autologous CFSE-labelled PBMC for a further 10 days. (A) Representative histograms show proliferating cells identified as CFSE^lo^ and the graph illustrates collated data (n=4; right; *P*=0.0992). (B) Dot plots show the frequencies of proliferating cells expressing CD3 and the graph shows collated data (*P*=0.5613). (C) Dot plots show the frequencies of proliferating T cells expressing CD4 or CD8 and the graphs show collated data (*P*=0.0663 and 0.0776, respectively). Statistically significant differences between DMSO- and SAHA-treated groups were determined by paired t-test, however, results were not statistically significant.

**Supplemental Figure 4: Supporting data for AM co-culture.** Human AM were adherence purified from BAL fluids and infected with H37Ra. After 24 hours, AM were washed and co-cultured with CFSE-labelled PBMC from a BCG-vaccinated healthy donor (IGRA negative) who responds to PPD antigens *in vitro*. Uninfected AM co-cultured with PBMC and Mtb-infected AM that were not co-cultured with PBMC were assayed in parallel as controls. (A) Representative graphs show the concentration of GM-CSF, TNF and IL-10 present in the supernatants on day 5 post co-culture; error bars indicate mean ±SD. (B) On day 10 post co-culture, PBMC were removed, stained with fluorochrome-conjugated antibodies specific for CD3, CD8, CD4, CD25 and intranuclear FoxP3 and analysed by flow cytometry. Representative dot plots/histograms show the gating strategy to identify Treg cells. (C) Graph illustrates collated data (n=4) for the frequencies of proliferating PBMC that are CD4^+^ Treg cells. (D-F) PBMC were isolated from the venous blood of the BCG-vaccinated healthy donor (IGRA negative) who responds to PPD antigens *in vitro*. MDM were adherence purified and infected with H37Ra in the presence of SAHA or vehicle control (DMSO). After 24 hours, MDM were washed and co-cultured with autologous CFSE-labelled PBMC. (D) The concentrations of IFN-γ present in the supernatants on the indicated days were analysed by ELISA; error bars indicate mean ±SEM of technical replicates. On day 10 post co-culture, PBMC were removed and stimulated with PMA/ionomycin in the presence of brefeldin A. Cells were stained with fluorochrome-conjugated antibodies specific for CD3, CD8, IFN-γ, TNF and GM-CSF and analysed by flow cytometry. (E) Dot plots show the co-staining of IFN-γ and GM-CSF within the proliferating CD4^+^ Th cell population. (F) MDM were lysed on day 0 (3 hours post infection), MDM alone and in co-culture were lysed on day 3 and day 10 (post co-culture with PBMC) and CFU were enumerated. Statistically significant differences between DSMO and SAHA treated groups were determined by two-way ANOVA with Bonferroni’s multiple comparisons test; **** *P*<0.0001.
